# The association between MMP-1 gene rs1799750 polymorphism and knee osteoarthritis risk

**DOI:** 10.1042/BSR20181257

**Published:** 2018-09-19

**Authors:** Rui Geng, Yuansheng Xu, Wenhao Hu, Hui Zhao

**Affiliations:** 1 Department of Orthopaedics, Yancheng Third People’s Hospital, The Affiliated Yancheng Hospital of Southeast University Medical College, Yancheng 224000, China; 2 Department of Orthopaedics, Bayi Hospital Affiliated to Nanjing University of Chinese Medicine, Nanjing 210002, China; 3 Department of Orthopaedics, Huaian First People’s Hospital, Nanjing Medical University, Huaian, China; 4 Department of General Surgery, Third Affiliated Hospital of Nantong University, Wuxi 214000, China

**Keywords:** knee OA, MMP-1, Single Nucleotide Polymorphism

## Abstract

Matrix metalloproteinase 1 (MMP-1) degrades cartilage, which may result in osteoarthritis (OA) development. Several studies have explored the association between *MMP-1* gene rs1799750 polymorphism and OA in different populations. However, the results are inconsistent. The aim of this case–control study was to investigate the association between MMP-1 gene rs1799750 polymorphism and knee OA in a Chinese population. The present study included 308 cases and 404 controls. Genotyping was performed using standard polymerase chain reaction and restriction fragment length polymorphism. The present study found that 2G2G genotype (2G2G vs 1G1G: OR & 95% CI, 2.28 (1.47–3.53), *P*<0.001; 2G2G + 1G2G vs 1G1G: OR & 95% CI, 1.61 (1.15–2.24), *P*=0.005; 2G2G vs 1G2G + 1G1G: OR & 95% CI, 1.84 (1.26–2.68), *P*=0.002) or 2G allele carriers (2G vs 1G: OR & 95% CI, 1.48 (1.20–1.83), *P*<0.001) of *MMP-1* gene rs1799750 polymorphism increased the risk of OA. In conclusion, this case–control study confirms that *MMP-1* gene rs1799750 polymorphism increases the risk of knee OA in Chinese Han population.

## Introduction

Osteoarthritis (OA), the most common type of arthritis, could cause progressive loss of joint function [1]. OA is characterized by biochemical, morphological, molecular, and biomechanical changes in both extracellular matrix (ECM) and cells, which lead to a softening, ulceration, fibrillation, sclerosis of subchondral bone, loss of articular cartilage, subchondral cysts, and osteophytes. OA is a combined result of environmental and genetic factors, while genetic factors account for nearly 50% of the risk of OA development [2]. Previous genome-wide association studies [3–5] have suggested that polymorphisms in certain genes affect the pathogenesis of OA. Therefore, candidate gene studies may provide insight for OA development.

*Matrix metalloproteinase 1* (*MMP-1*) gene, located on the long arm of chromosome 11 (11q22.3), is expressed in various cells, such as chondrocytes, fibroblasts, epithelial and endothelial cells, and tumor cells [6]. Rs1799750 is an important intron variant of *MMP-1* gene. The global minor allele frequency (MAF) of this single nucleotide polymorphism (SNP) was 0.473 according to the data of dbSNP database. MMP-1 is produced by synovial cells, chondrocytes, and osteoblasts, which can degrade ECM collagen and mediate cartilage destruction [7,8]. Expression of MMP-1 is low in normal cells that leads to healthy cartilage remodeling. In pathological conditions, the level of MMP-1 expression increases considerably, resulting in aberrant connective tissue destruction [9]. MMP-1 is expressed at higher levels in OA chondrocytes than in normal chondrocytes, suggesting a predominant role of MMP-1 in OA pathogenesis [10].

Several studies reported the association between *MMP-1* gene rs1799750 polymorphism and the risk of OA [11–15]. However, the results are contradictory. Thus, we conduct a case–control study in a Chinese Han population to investigate the relationship between *MMP-1* gene rs1799750 polymorphism and knee OA risk.

## Materials and methods

### Patients

A hospital-based case–control design was used in the present study. A total of 308 knee OA patients were selected from the Huaian First People’s Hospital. The diagnosis of OA in all patients was based on the criteria of the American College of Rheumatology, which included primary OA with any symptoms and signs of OA, and radiographic signs of OA according to the Kellgren–Lawrence grading. Other etiologies of OA, including inflammatory arthritis, post-traumatic or post-septic arthritis, skeletal dysplasia, or developmental dysplasia were excluded.

The control group comprised a total of 404 individuals randomly selected from among subjects who received regular health examinations at Huaian First People’s Hospital between January 2013 and October 2017. The control group never had any signs or symptoms of OA, other arthritis or joint diseases (pain, swelling, tenderness, or restriction of movement) at any site based on their medical history and a thorough examination conducted by an experienced physiatrist. The control subjects had no relationship with the patients and no family history of OA.

The demographic, lifestyle, and clinical characteristics of all patients and controls, such as gender, age, body mass index (BMI), and Kellgren–Lawrence grading were collected from medical records. Written informed consent was obtained from all included patients and controls. We obtained approval for the study protocol from the Ethics Committee of Huaian First People’s Hospital. The ethical approval of our study was in line with the standards of the Declaration of Helsinki.

### DNA extraction and genotyping

Blood samples were collected using vacutainer tubes and transferred to EDTA tubes. Genomic DNA was isolated from whole blood using the QIAamp DNA Blood Mini Kit (Qiagen, Hilden, Germany). Genotyping was carried out by standard polymerase chain reaction and restriction fragment length polymorphism (PCR-RFLP). PCRs were performed in 25 ml volume containing 100 ng of DNA template, 2.5 ml of 10× PCR buffer, 2.0 mM MgCl_2_, 2.5 U of Taq-DNA-polymerase (LifeTechnologies, Inc.), 0.2 mM dNTPs (Sigma Chemical Co.), and 0.2 mM primers (Biosynthesis). Amplification was performed using a PTC-100 thermal cycler (MJ Research, Inc.). The PCR cycling conditions were 2 min at 95°C followed by 35 cycles of 45 s at 94°C, 60 s at 49.5°C (MMP1), and 60 s at 72°C, and with a final extension at 72°C for 10 min. PCR products were digested for 16 h at 37°C (MMP-1) in a 15 ml reaction containing 5 U *AluI* restriction enzyme (New England Biolabs) for determination of MMP-1 genotypes. The digested products were subjected to gel electrophoresis and visualized by ethidium bromide staining. To control the quality of genotyping, the PCR-RFLP method was performed without knowing the status of the cases or controls.

### Statistical analysis

Student’s *t*-test and Chi-squared (χ^2^) test were used to evaluate the differences in demographics, variables, and genotypes of the *MMP-1* gene rs1799750 polymorphism variants. The associations between *MMP-1* gene rs1799750 polymorphism 1G/2G genotypes and the risk of knee OA were estimated by computing the odds ratios (ORs) and 95% confidence intervals (CIs) using logistic regression analysis. The Hardy–Weinberg equilibrium (HWE) was tested by a goodness-of-fit χ^2^ test to compare the observed genotype frequencies to the expected ones among controls. Above statistical analyses were performed using the SAS software package (version 9.1.3; SAS Institute, Cary, NC, U.S.A.). The power of the present study was calculated with a significant value of 0.05 [16].

## Results

### Characteristics of the study population

The characteristics of the subjects in the case and control groups are summarized in Table 1. The average age was 51.42 years in the case groups and 46.1% of knee OA patients were women. In the control groups, the average age was 51.64 years and 50.5% were men. Cases and controls were well matched in terms of gender, age, and BMI. No significant differences in gender, age, and BMI were observed between the OA patients and controls. Kellgren–Lawrence grading were listed in Table 1.

**Table 1:** Subjects demographics and risk factors in knee OA

Variable	Cases (*n*=308)	Controls (*n*=404)	*P*
Sex			0.245
Male	166 (53.9%)	200 (49.5%)	
Female	142 (46.1%)	204 (50.5%)	
Age (years)	51.42 ± 6.69	51.64 ± 7.02	0.670
BMI (kg/m^2^)	24.40 ± 1.45	24.25 ± 1.54	0.185
ESR (mm/h)	13.41 ± 4.35	13.80 ± 4.27	0.227
CRP (mg/l)	2.58 ± 0.77	1.69± 0.44	<0.001
Kellgren–Lawrence grading			
1	66 (21.5%)	–	–
2	147 (47.6%)	–	–
3	87 (28.3%)	–	–
4	8 (2.6%)	–	–

### Association between *MMP-1* gene rs1799750 polymorphism and knee OA risk

The genotype distributions of *MMP-1* gene rs1799750 polymorphism in all subjects are delineated in Table 2. Genotype distributions for rs1799750 polymorphism in the controls conformed to the HWE. Logistic regression analyses revealed that 2G2G genotype (2G2G vs 1G1G: OR & 95% CI, 2.28 (1.47–3.53), *P*<0.001; 2G2G + 1G2G vs 1G1G: OR & 95% CI, 1.61 (1.15–2.24), *P*=0.005; 2G2G vs 1G2G + 1G1G: OR & 95% CI, 1.84 (1.26–2.68), *P*=0.002) or 2G allele carriers (2G vs 1G: OR & 95% CI, 1.48 (1.20–1.83), *P*<0.001) of *MMP-1* gene rs1799750 polymorphism increased the risk of OA (Table 2). Moreover, several significant associations were observed (Female: 2G2G vs 1G1G, OR & 95% CI, 3.13 (1.65–5.96), *P*<0.001; 1G1G + 1G2G vs 2G2G, OR & 95% CI, 2.56 (1.48–4.45), *P*<0.001; 1G1G vs 1G2G + 2G2G, OR & 95% CI, 1.72 (1.06–2.79), *P*<0.027; Age <55: 2G2G vs 1G1G, OR & 95% CI, 2.14 (1.27–3.60), *P*=0.004; 1G1G + 1G2G vs 2G2G, OR & 95% CI, 1.82 (1.16–2.85), *P*=0.009; 1G1G vs 1G2G + 2G2G, OR & 95% CI, 1.50 (1.01–2.23), *P*=0.045; Age ≥55: 2G2G vs 1G1G, OR & 95% CI, 2.61 (1.16–5.89), *P*=0.020; 1G1G vs 1G2G + 2G2G, OR & 95% CI, 1.87 (1.03–3.42), *P*=0.041) between genotypes and the clinical and biochemical characteristics (Table 3).

**Table 2:** Logistic regression analysis of associations between rs1799750 polymorphism and risk of knee OA

Genotype	Cases* (*n*=308)		Controls* (*n*=404)		OR (95% CI)	*P*
	*n*	%	*n*	%		
1G2G vs 1G1G	154/76	50/24.7	201/139	49.8/34.4	1.40 (0.98–1.99)	0.059
2G2G vs 1G1G	76/76	24.7/24.7	61/139	15.1/34.4	**2.28 (1.47–3.53)**	<0.001
2G2G + 1G2G vs 1G1G	230/76	74.7/24.7	262/139	64.9/34.4	**1.61 (1.15–2.24)**	0.005
2G2G vs 1G2G + 1G1G	76/230	24.7/74.7	61/340	15.1/84.2	**1.84 (1.26–2.68)**	0.002
2G vs 1G	306/306	49.7/49.7	323/479	40.0/59.3	**1.48 (1.20–1.83)**	<0.001

Bold values are statistically significant (*P*<0.05).

* The genotyping was successful in 308 cases and 404 controls.

**Table 3:** The clinical and biochemical characteristics of rs1799750 polymorphism among two groups

	rs1799750 (case/control)			1G2G vs 1G1G	2G2G vs 1G1G	1G1G + 1G2G vs 2G2G	1G1G vs 1G2G + 2G2G
Variable	1G1G	1G2G	2G2G				
Sex							
Male	42/68	85/96	37/35	1.43 (0.89–2.32); 0.144	1.71 (0.94–3.12); 0.080	1.37 (0.81–2.29); 0.238	1.51 (0.96–2.38); 0.078
Female	34/71	69/105	39/26	1.37 (0.83–2.28); 0.223	**3.13 (1.65–5.96); <0.001**	**2.56 (1.48–4.45); <0.001**	**1.72 (1.06–2.79); 0.027**
Age							
<55	55/92	103/133	55/43	1.29 (0.85–1.98); 0.229	**2.14 (1.27–3.60); 0.004**	**1.82 (1.16–2.85); 0.009**	**1.50 (1.01–2.23); 0.045**
≥55	21/47	51/68	21/18	1.68 (0.89–3.15); 0.107	**2.61 (1.16–5.89); 0.020**	1.86 (0.93–3.73); 0.079	**1.87 (1.03–3.42); 0.041**

Bold values are statistically significant (*P*<0.05).

We further made analysis between clinical and biochemical characteristics of the two OA groups and MMP1 gene rs1799750 polymorphism. However, no significant findings were obtained (Table 4). Last but not least, we found the power of the present study reach a value of 73.2%, with an OR of 2.28.

**Table 4:** The clinical and biochemical characteristics of MMP1 rs1799750 polymorphism among two groups.

	Patients(*n*=308)				Controls (*n*=404)			
	1G1G (*n*=76)	1G2G (*n*=154)	2G2G (*n*=76)	*P*	1G1G (*n*=139)	1G2G (*n*=201)	2G2G (*n*=61)	*P*
BMI (kg/m^2^)	24.38 ± 1.49	24.37 ± 1.47	24.48 ± 1.38	0.862	24.38 ± 1.41	24.14 ± 1.64	24.27 ± 1.57	0.348
ESR (mm/h)	13.73 ± 4.16	13.16 ± 4.37	13.62 ± 4.57	0.585	14.29 ± 4.07	13.49 ± 4.29	13.56 ± 4.57	0.209
CRP (mg/l)	2.61 ± 0.79	2.57 ± 0.76	2.56 ± 0.79	0.901	1.74 ± 0.46	1.65 ± 0.43	1.70 ± 0.40	0.144

Bold values are statistically significant (*P*<0.05).

## Discussion

In the present study, we investigated the association between *MMP-1* gene rs1799750 polymorphism and the risk of knee OA in a Chinese population and found that the *MMP-1* gene rs1799750 polymorphism may increase the risk of knee OA.

MMPs have been associated with the pathological destruction of joint tissues in OA [17]. In recent years, some functional polymorphisms of MMPs, including *MMP-1* gene rs1799750 polymorphism, have been identified. Rutter et al. [18] indicated that 2G allele rs1799750 polymorphism in *MMP-1* gene may result in an increased transcriptional activity and MMP-1 expression. This SNP was investigated in various diseases. Jacobsen et al. [19] showed that *MMP-1* rs1799750 2G allele was associated with increased low back pain, sciatica, and disability after lumbar disk herniation. They also indicated that rs1799750 polymorphism increased the MMP1 expression *in vitro* [19]. Rech et al. [20] uncovered that *MMP-1* gene variants were associated with systemic sclerosis. A host of studies also demonstrated that *MMP-1* gene rs1799750 polymorphism was related to cancer susceptibility, such as leukemia [21] and gastric cancer [22]. We assumed that rs1799750 polymorphism mediated the MMP-1 expression, thereby contributing to the risk for many diseases, which needs further studies to verify it.

Recently, many studies have explored the association between *MMP-1* gene rs1799750 polymorphism and OA risk, but with conflicting results. Barlas et al. [11] first suggested that the rs1799750 polymorphism in *MMP-1* gene may contribute to susceptibility to knee OA in the Turkish population. Abd-Allah et al. [12] also reported that *MMP-1* gene rs1799750 polymorphism increased the OA risk in an Egyptian population. Lepetsos et al. [13] observed no significant association between *MMP-1* gene rs1799750 polymorphism and knee OA in a Greek study. As for Asian populations, Luo et al. [14] found that rs1799750 polymorphism of *MMP-1* gene increased the risk of temporomandibular joint OA susceptibility in a Chinese population while Yang et al. [15] suggested no association between this SNP and knee OA. It is obvious that these abovementioned Caucasian and Asian studies obtained inconsistent findings. The following factors may contribute to it. Different geographical environments, sample sizes, and study designs may explain these discrepancies. For example, in the Chinese case–control study [15], age and gender-matched control subjects were selected at random from the hospital-based population. In contrast, controls were not matched for age and sex from the Turkish study [11]. Cases in the Egyptian study were considerably younger than cases in the Chinese study [15] (mean age of the Egyptian case group was 44 years, compared with 51 years of the Chinese cases). Moreover, cases included in the Greek study [13] were adults who had undergone total knee replacement with a radiological score >2 in Kellgren–Lawrence scale while the cases included in other studies just met the diagnostic criteria of OA, which were regarded as different clinical heterogeneity. Another point that could not be ignored was that the types of OA were different, such as mixed OA, knee OA, and temporomandibular joint OA, which may also be a factor for contradictory findings.

Several potential limitations of this case–control study should be considered. First, a single case–control study may not be sufficient to fully interpret the relationship between the *MMP-1* gene rs1799750 polymorphism and knee OA susceptibility because of the relatively small number of subjects involved. Larger sample sizes of different case–control studies are necessary to confirm our findings. Second, the patients and controls were enrolled from hospitals and may not represent the general population, although the genotype distribution of the controls was in HWE. Third, we did not obtain detailed information about OA severity and response to treatment, which restricted our analyses of the role of *MMP-1* gene rs1799750 polymorphism in OA. Finally, the results of the Chinese population may not generalize to other ethnic groups, so further studies in different populations are warranted.

In conclusion, the present study provides strong evidence that *MMP-1* gene rs1799750 polymorphism may contribute to the risk of OA. However, our results were obtained with a limited sample size, and therefore, this is a preliminary conclusion. Validation by more multicenter case–control studies from diverse ethnic populations is needed to confirm our finding.

## Abbreviations

BMI: body mass index
CI: confidence interval
CRP: C reactive protein
ECM: extracellular matrix
ESR: erythrocyte sedimentation rate
HWE: Hardy–Weinberg equilibrium
MAF: minor allele frequency
MMP-1: matrix metalloproteinase 1
dNTP: deoxy-ribonucleoside triphosphate
OA: osteoarthritis
OR: odds ratio
PCR-RLFP: polymerase chain reaction and restriction fragment length polymorphism
SNP: single nucleotide polymorphism
